# I will teach you here or there, I will try to teach you anywhere: perceived supports and barriers for emergency remote teaching during the COVID-19 pandemic

**DOI:** 10.1186/s40594-022-00335-1

**Published:** 2022-02-19

**Authors:** Cristine Donham, Hillary A. Barron, Jourjina Subih Alkhouri, Maya Changaran Kumarath, Wesley Alejandro, Erik Menke, Petra Kranzfelder

**Affiliations:** 1 Department of Genetics, University of Georgia, Athens, GA USA; 2 Biology Teaching and Learning, University of Minnesota, Minneapolis, MN USA; 3 Quantitative and Systems Biology Program, University of California, Merced, CA USA; 4 Cognitive and Information Sciences, University of California, Merced, CA USA; 5 Molecular and Cellular Biology, University of California Merced, Merced, CA USA; 6 Chemistry and Biochemistry, University of California, Merced, CA USA; 7 Department of Biology, Bemidji State University, Bemidji, MN USA

**Keywords:** Qualitative, STEM, Undergraduate, Instructor, Minority-Serving Institution

## Abstract

**Background:**

Due to the COVID-19 pandemic, many universities moved to emergency remote teaching (ERT). This allowed institutions to continue their instruction despite not being in person. However, ERT is not without consequences. For example, students may have inadequate technological supports, such as reliable internet and computers. Students may also have poor learning environments at home and may need to find added employment to support their families. In addition, there are consequences to faculty. It has been shown that female instructors are more disproportionately impacted in terms of mental health issues and increased domestic labor. This research aims to investigate instructors’ and students’ perceptions of their transition to ERT. Specifically, during the transition to ERT at a research-intensive, Minority-Serving Institution (MSI), we wanted to: (1) Identify supports and barriers experienced by instructors and students. (2) Compare instructors’ experiences with the students’ experiences. (3) Explore these supports and barriers within the context of *social presence*, *teaching presence*, and/or *cognitive presence* as well as how these supports and barriers relate to *scaffolding* in STEM courses.

**Results:**

Instructors identified twice as many barriers as supports in their teaching during the transition to ERT and identified casual and formal conversations with colleagues as valuable supports. Emerging categories for barriers consisted of academic integrity concerns as well as technological difficulties. Similarly, students identified more barriers than supports in their learning during the transition to ERT. More specifically, students described pre-existing course structure, classroom technology, and community as best supporting their learning. Barriers that challenged student learning included classroom environment, student availability, and student emotion and comfort.

**Conclusions:**

Together, this research will help us understand supports and barriers to teaching and learning during the transition to ERT. This understanding can help us better plan and prepare for future emergencies, particularly at MSIs, where improved communication and increased access to resources for both students and instructors are key.

**Supplementary Information:**

The online version contains supplementary material available at 10.1186/s40594-022-00335-1.

## Introduction

In the middle of the Spring 2020 academic term, many institutions of higher education were forced to move all instruction online. The term “pandemic pedagogy” was quickly coined as educators, many of whom had never taught online or remotely, scrambled to come up with effective ways to teach their courses (Schwartzman, 2020). Although moving to remote instruction can enable flexibility of teaching and learning (Daymont et al., 2011), the speed at which instructors and students were expected to move to remote instruction was unprecedented. Therefore, it is important to distinguish this quick transition to remote teaching, or *emergency remote teaching* (ERT), from the traditional *online teaching and learning*. Here, we use the term *online teaching and learning* to refer to traditional online teaching (i.e., teaching online during non-pandemic times), and have adopted Charles Hodges’s definition of ERT as a temporary shift of instructional delivery to an alternate delivery mode due to crisis circumstances (Hodges et al., 2020). ERT is characterized by improvised, quick solutions in less-than-ideal circumstances, and it was the best solution most universities had to academic learning. This is different from traditional *online teaching and learning,* where instructors are intentionally designing a course to be implemented and delivered online, a delivery mode that has been studied for decades (e.g., Bender, 2012; Lewis & Abdul-Hamid, 2006; Oliver, 1999; Young, 2006). There are numerous research studies, theories, models, and evaluation criteria created for traditional *online teaching and learning* (e.g., Oliver, 2000; Ouyang & Scharber, 2018; Shelton & Hayne, 2017). Studies have shown that effective online learning stems from careful instructional design, planning, and using a systematic model for development (Branch & Kopcha, 2014). This careful design process was likely to be absent in most ERT shifts due to lack of time and experience necessary for instructors to carefully design their course for online purposes.

In addition to a shortage of time and experience, the move to ERT introduced a variety of issues that instructors and students had not faced during in-person teaching, such as lack of communication (Gelles et al., 2020). Previous research on emergency teaching during Hurricane Katrina in 2005 showed that virtual student-to-student interactions and remote class dialogues created opportunities for students to provide mental and emotional support for each other (Lorenzo, 2008). Other issues with ERT, include navigating the course in a new manner, finding new ways to implement formative assessment, communicating with students in a fair and equitable manner, monitoring academic integrity, and managing everything through a remote platform (Brooks & Grajek, 2020; Johnson et al., 2020). In addition, many students moved home and were expected to attend college from home while being quarantined with their family. Students needed to learn how to navigate a new learning platform and adjust to new patterns, all while experiencing loss of social interactions and community. Shay and Pohan (2021) described how “many low-income, first-generation students also struggle with a lack of quiet workspaces, the absence of internet and other technological tools, housing and/or food insecurity, and the added responsibilities associated with being at home and helping the family (e.g., employment, care, etc.).” Stress, anxiety, and traumatic events contributed to students’ cognitive load, the demand or burden on one’s working memory, making the focus on learning more challenging for students (Shay & Pohan, 2021). Finally, high-quality internet is an enabling technology for ERT, and in the United States approximately 21 million people, or 6.5% of the population, did not have broadband internet access (Chavez, 2020; Commission, 2018). This lack of access to technology during ERT created a digital divide in higher education for both instructors and students.

Since ERT began as a result of COVID-19, there have been new studies examining a wide variety of impacts on students and instructors (Affouneh et al., 2020; Bozkurt & Sharma, 2020; Iglesias-Pradas et al., 2021; Karakaya, 2021; Whalen, 2020). For example, Iglesias-Pradas et al. (2021) explored the move to ERT and the impacts of organizational aspects related to unplanned changes as well as instruction-related variables, such as class size, synchronous versus asynchronous instruction, and digital supporting technologies used on students’ academic performance. They found that the students’ academic performance increased and discussed how this supports the idea that organizational factors could contribute to successful implementation of ERT. They also found that there were no differences in academic performance across different class sizes and delivery models. Trust and Whalen (2020) looked at whether instructors should be trained for ERT by administering a survey to learn more about educator’s experiences. The surveys revealed that the instructors surveyed felt overwhelmed and unprepared to use remote teaching strategies and tools and they needed more support when shifting their practices. Finally, Wester et al. (2021) examined how the shift in learning environment for students impacted student engagement and found that student engagement significantly decreased during ERT.

Despite this new literature, there is a need to further understand the effects of ERT by examining what helped and hindered both instructors and students during their transition to ERT. This is of particular importance at institutions, where students are more at risk of being disproportionately affected due to their background and socioeconomic status, especially students attending Minority-Serving Institutions (MSIs). We aim to help fill this gap by studying supports and barriers during the transition to ERT at a research-intensive, MSI, the University of California Merced (UC Merced).

UC Merced is in a unique situation to study the rapid transition to ERT. While it is a member of a well-resourced university system (i.e., UC system), its student population is highly diverse and like student populations that are typically understudied in education research (Kanim & Cid, 2020; National Research Council, 2012). For example, during the 2019–2020 academic year the UC Merced undergraduate population was 74% first-generation, 54% Hispanic (9% non-Hispanic white), and 64% Pell-Grant eligible. This student population is representative of populations across the country that are disproportionately being impacted by COVID-19, as they are vulnerable to living in persistent poverty, holding unstable or uncertain employment, and without stable housing (Burke et al., 2020).

In addition, UC Merced’s faculty population is younger (82.3% of UC Merced STEM faculty under age 55, compared to 65.6% across the UC system) and more likely to be a woman (34.3% of UC Merced STEM faculty are women, compared to 30.7% across the UC system) (University of California, 2020, 2021. Like the student population, the faculty population is more likely to suffer personally and professionally from the negative impacts of the COVID-19 pandemic than the broader UC system population. Female instructors in general, are thought to be more disproportionately impacted in terms of mental health issues and increased domestic labor due to the pandemic (Brooks & Grajek, 2020; Donner, 2020; Krentz et al., 2020). Because of these factors, we wanted to understand the supports and barriers experienced by UC Merced instructors and students during the transition to ERT.

## Theoretical frameworks

Our work is guided by the *Community of Inquiry* (COI) (Garrison et al., 2010a) and the *scaffolding* (Wass et al., 2011) frameworks. First, the COI framework is a collaborative–constructivist process model that describes the essential elements of successful online higher education learning experiences (Garrison, 2016; Garrison et al., 1999). The COI framework has been widely used when investigating traditional *online teaching* as it is grounded in a social constructivist approach to learning (e.g., Arbaugh et al., 2008; Castellanos-Reyes, 2020; Garrison et al., 1999; Piaget, 1976). In addition, drawing from this social constructivist lens, learners co-construct knowledge by engaging in actions that elicit and validate their sociocultural identities (Dewey, 1986; Vygotsky, 1978). Vygotsky contended that socially situated learning, accompanied by *scaffolding*, led to stronger outcomes in both knowledge development and retrieval (Dewey, 1986; Vygotsky, 1978). Equity-driven pedagogies in STEM education are grounded in constructing learning experiences for students that foster agency (Dewsbury & Brame, 2019). In the rapidly shifting realities of pandemic-induced ERT, instructor resources and ability to create socially situated learning environments were uncertain, as was student accessibility to online teaching. In addition to the COI framework, we utilized the *scaffolding* framework as a collaborative–constructivist process model from which we examined how instructors have adapted to remote pedagogy and the supports and barriers that instructors and students found helped them or hindered them. *Scaffolding* refers to the help or guidance from a more competent peer or mentor that allows students to work within, and then move beyond, the zone of proximal development (ZPD) (Wass et al., 2011).

### Community of Inquiry

**Fig. 1:**
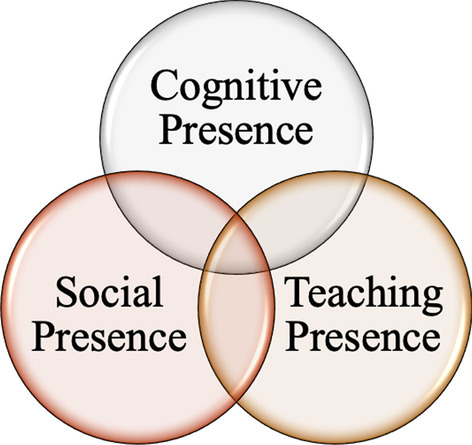
Community of Inquiry (COI). The COI theoretical framework represents a process of creating a deep and meaningful, collaborative-constructivist, learning experience through the development of three interdependent elements – social, cognitive, and teaching presence ( adapted from Anderson et al., 2001)

In general, the COI framework is a process model of online learning which views the online educational experiences as arising from the interaction of three elements: *teaching presence, social presence*, and *cognitive presence* (Fig. 1). In the COI framework, *teaching presence*, is defined as the design, facilitation, and direction of cognitive and social processes to realize teacher-defined learning outcomes. This element has three components: (1) instructional design and organization (e.g., instructor provides clear instructions on how to achieve course learning outcomes); (2) facilitating discourse (e.g., instructor helps to keep course participants engaged and participating in productive dialogue); and (3) direct instruction (e.g., instructor presents useful examples that allows students to better understand course content) (Anderson et al., 2001). Research has shown that *teaching presence* is important for successful online learning and strongly correlates with student satisfaction, perceived learning, and sense of community (Kucuk & Richardson, 2019; Liu, 2019; Manwaring et al., 2017; Soffer & Cohen, 2019). Shea and Bidjerano (2008) found that the quality of *teaching presence* and *social presence* reported by learners in online courses could predict learning represented by the *cognitive presence*. In addition, Garrison, et al. (2010) found that student perceptions of *teaching presence* predicted significant direct effect on perceptions of *cognitive presence,* while *social presence* had an indirect or mediating effect on *cognitive presence*. Therefore, *teaching presence* has been shown to be central in creating quality online learning experiences.

The second element in the COI framework, *social presence*, is defined as the ability for learners to be perceived as “real people.” It is primarily focused on affective expression (e.g., students perceived online or web-based communication as an excellent medium for social interaction), open communication (e.g., students felt comfortable interacting with other course participants), and group cohesion (students perceived online discussions as helping them develop a sense of collaboration). Research on *social presence* has demonstrated a strong relationship between *social presence* and student learning outcomes (d’Alessio et al., 2019; Hwang & Arbaugh, 2006; Richardson et al., 2017). In addition, research has shown that activities that cultivate *social presence* also enhance learner satisfaction (Richardson et al., 2017) and that interaction and engagement through active learning is necessary for students to feel as they are dealing with real people, that they belong in some way to a group of learners, and that they are involved in sharing, negotiating, arguing, and discussing (Wang, 2008). In general, online learning environments should be active, allow student to construct their own knowledge, make effective use of collaborative and cooperative learning methods, and be meaningful to students while promoting *social presence* and community (Ally, 2004).

Finally, the third element, *cognitive presence*, is defined as the extent to which learners can construct and confirm meaning through sustained reflection and discourse. It is rooted in social constructivism, which is a robust theory proposing that people’s learning is shaped by cultural context, conversation, and collaboration (Dewey, 1986; Vygotsky, 1978). *Cognitive presence* has four components: (1) triggering event (e.g., students felt motivated to explore content related questions); (2) exploration (e.g., students used a variety of information sources to explore problems posed in the course); (3) integration (e.g., learning activities helped students construction explanations/solutions); and (4) resolution (e.g., students applied the knowledge created in the course to their work and non-class related activities). Of the three elements in COI, *cognitive presence* has been identified as the most difficult to study as well as the most challenging to develop and sustain in online courses (Garrison & Cleveland-Innes, 2005). This difficulty arises from the fact that *cognitive presence* contains inputs (the triggering event), processes (exploration and integration), and outputs (resolution) that can be hard to measure or observe, whereas the other two elements consist of processes that can be more easily observed (Garrison & Arbaugh, 2007).

While the COI framework is valuable for understanding online learning, it has only recently been used to understand ERT. For example, it forms the foundation for recent research on K-12 teachers during ERT (Whittle et al., 2020), Reinholz et al. (2020) used the COI framework to study how the nature of student participation changed in moving from face-to-face to synchronous online learning environments at an Hispanic-Serving Institution (HSI), and Erickson and Wattiaux (2021) studied how student perceptions of *social presence*, *cognitive presence*, and *teaching presence* online due to COVID-19 were influenced by student demographics. This research has been in response to the COVID-19 pandemic and demonstrates that there are some shortcomings for the COI framework as a means to understand ERT. In particular, the crisis nature of a global pandemic, such as rapidly shifting personal responsibilities, the emotional toll of uncertain life situations, and the changing ability to access information, led to an increased difficulty with implementing online instruction. This was of particular importance for students with fewer resources and weaker safety nets, such as those at MSIs. This implementation difficulty, coupled with time constraints of a rapid shift from in-person teaching to ERT, can be expected to lead to lower *teaching presence*, *social presence*, and *cognitive presence* in ERT when compared to online teaching. While there have been alternative frameworks proposed to evaluate ERT, such as the Emergency Remote Teaching Environment framework (ERTE) proposed by Whittle et al. (2020), we have chosen to use the COI framework, because we are primarily interested in why the *teaching*, *social*, and *cognitive presence* might be different during ERT compared to online teaching.

### Scaffolding in distance learning

In 1976, Wood, Bruner and Ross introduced the term *scaffolding* (Wood et al., 1976) and many researchers and educators have used the concept of *scaffolding* to describe instructor roles as more knowledgeable peers for guiding student learning and development (Hammond, 2001; Stone, 1998; Wells, 1999). Scaffolding has been interpreted in a wide sense as “a form of support for development and learning” (Rasmussen, 2001, p. 570). Alternatively, it can be used as an umbrella metaphor to describe the way that teachers supply students with the tools necessary to learn (Jacobs, 2001).

**Fig. 2:**
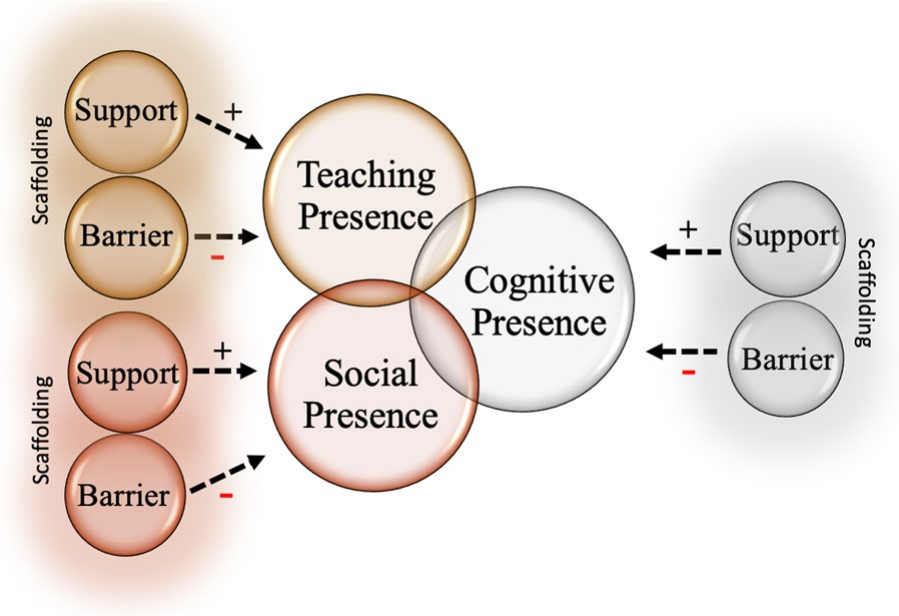
The Community of Inquiry and Scaffolding. Each support and barrier influenced its designated presence/attribute and each presence/attribute ultimately led to a specific *cognitive presence* for instructors and students. ( +) indicate positive influences in each presence or attribute and (–) indicates negative influences. Scaffolding is represented as the glow around each support and barrier as it impacts both

While there are different approaches in the literature on how *scaffolding* may or may not be intertwined with Vygotsky’s ZPD (Vygotsky, 1980), we used Wells’ argument that *scaffolding* can be a direct application and operationalization of ZPD (Berk, 2003; Duchesne & McMaugh, 2018; Wells, 1999). Wells identified three features that characterize educational *scaffolding*: (1) The essentially dialogic nature of the discourse in which knowledge is co-constructed (dialog); (2) the significance of the kind of activity in which knowing is embedded (activity); and (3) the role of artifacts that mediate knowing (artifacts) (Wells, 1999, p. 127). Furthermore, the relationship between classroom challenge (or barrier) and support is important in *scaffolding* (Hammond & Gibbons, 2005). Hammond and Gibbons (2005) found that highly supportive, but minimally challenging environments (or low in barriers) may be too easy to elicit growth in knowledge, whereas experiences that are highly challenging but lack sufficient support will likely result in failure (Fig. 2). This becomes an important aspect of *scaffolding* to consider as we examine the supports and barriers that instructors and students experienced during the transition through ERT. Therefore, during the transition to ERT at a research-intensive, MSI, the three objectives of this study were to:

1. Identify supports and barriers experienced by instructors and students during the transition to ERT.
2. Compare the instructors’ experience with the students’ experiences.
3. Explore these supports and barriers within the context of *social presence*, *teaching presence*, and/or *cognitive presence* as well as how these supports and barriers relate to *scaffolding* in STEM courses.

## Methods

### Recruitment

The study was approved by the Human Subjects Committee of the University of California Merced’s Institutional Review Board (IRB) (Protocol ID UCM2020-3).

#### Instructors

In May 2020, we sent an initial recruitment email, via departmental list serves and individual email addresses, to tenure-track and non-tenure track faculty and graduate teaching assistants who were teaching STEM courses at UC Merced. UC Merced is a mid-sized, public, research-intensive university designated as an MSI in the UC system. The initial email included the purpose of the study, procedures, benefits, IRB approval, potential dissemination of the results, question information, and contact information. Instructor recruitment occurred approximately 2 months after the institutional switch to ERT.

We chose the instructors in this study due to their ongoing involvement in a larger institutional study assessing teaching and discourse practices in college STEM classrooms. This larger study focused on instructors who (1) taught either a lower or upper division undergraduate or graduate STEM course and (2) taught a lecture or laboratory course. To recruit participants for this study on the transition to ERT, we applied the same requirements (1 and 2) plus two additional criteria: (3) taught a remote course via synchronous instruction (excluded in-person and asynchronous instruction), and (4) taught during the Spring 2020 semester.

#### Students

During the instructor recruitment process, all instructors were asked if they would be willing to invite their students to a 30-min in-class or out-of-class group interview. Out of 31 instructors that agreed to participate in the instructor interviews, 22 agreed to inviting their students to these interviews (71% participation rate). Students from five courses attended and agreed to participate in these group interviews (16% participation rate).

### Population

We interviewed 31 instructors and surveyed 69 students. Instructors taught biology, chemistry, engineering, mathematics, and physics courses, while students were in biology, chemistry, and physics courses (Table 1). Gender and ethnically appropriate pseudonyms were created for instructors and gender–neutral pseudonyms were created for students to de-identify participants and retain their privacy and confidentiality.

**Table 1:** Demographics of instructors (Faculty and TAs) (*n* = 31) and students (n = 69)

STEM discipline	*Instructors*	*Students*
Biology	15	1
Chemistry	8	31
Engineering	4	0
Mathematics	3	0
Physics	1	37

### Data collection

#### Instructors

Instructor interviews were carried out via Zoom between May 11 and May 27, 2020. The semi-structured interviews ranged in length from 10 to 90 min, where the participants were asked six questions regarding perceived supports and barriers, changes in interactions with students and instructors, pedagogical changes made or planned, and potential future supports. Due to the nature of semi-structured interviews, there was a wide range of time lengths. This is partly because there were only a few questions that were planned (our 6 specific questions) and the rest of the questions asked were dependent on the interviewee’s responses. For example, if one interviewee was not particularly responsive or lengthy in their responses to questions, then this would create a short interview. This manuscript will focus only on perceived supports and barriers (i.e., questions 1–2 from Table 2).

**Table 2:** Interview questions

Instructor	Student
1. What has helped, what supported your teaching?	1. What has helped, what supported your learning?
2. What has challenged your teaching, what barriers did you face? What are your biggest concerns?	2. What has challenged your learning, what barriers did you face?
3. How did your interactions with your TAs (or instructor) change?	3. How did your interactions with the instructors (faculty, lecturer, TAs) change?
4. How did your interactions with your students change?	4. How did your interactions with other students change?
5. Are you aware of any pedagogical changes that you made? What effect do you believe these have on student learning? How have these changes impacted your approach to teaching?	5. What else did you need to succeed? What other supports did you need?
6. Will you keep any of these pedagogical changes for future semesters?	

Evidence based teaching (EBT) practices, including inclusion of formative assessments and active learning, have been shown to support student learning (Cavanagh et al., 2018; Freeman et al., 2014). Currently, there is a breadth of literature that focuses on perceived supports and barriers of college STEM instructors in their implementation of EBT practices (Bathgate et al., 2019a, b; Brownell & Tanner, 2012). This literature indicates that a focus on building supports for instructors may have the greatest impact on increased implementation of EBT (Bathgate et al., 2019a). Therefore, we chose to focus on perceived supports and barriers during ERT.

Instructors were not interviewed by anyone from their own department (i.e., biology faculty interviewed chemistry faculty). Four of the authors conducted the 31 interviews. After the interviews were completed, they were transcribed using a clean verbatim transcription service.

#### Students

Student surveys and group interviews were carried out via Zoom during the final week of instruction (between May 5 and May 8, 2020) by three Students Assessing Teaching and Learning (SATAL) interns, an assessment team of trained undergraduate students out of UC Merced’s Center for Teaching and Learning (CETL) (Signorini & Pohan, 2019). SATAL interns were used, because they allowed for data to be collected from students without an instructor being present, which created a more confidential and safer environment for the students. Individual surveys and group interviews lasted about 30 min during discussion sections, where the participants were asked five questions regarding perceived supports and barriers, changes in interactions with instructors and other students, and potential future supports. Before the start of the group interviews, students were asked to individually answer questions via a Qualtrics survey. This manuscript will focus only on perceived supports and barriers from the individual surveys, not group interviews, as they would be more representative of the student experience (Table 2).

### Data analyses

#### Instructor interview coding

**Fig. 3:**
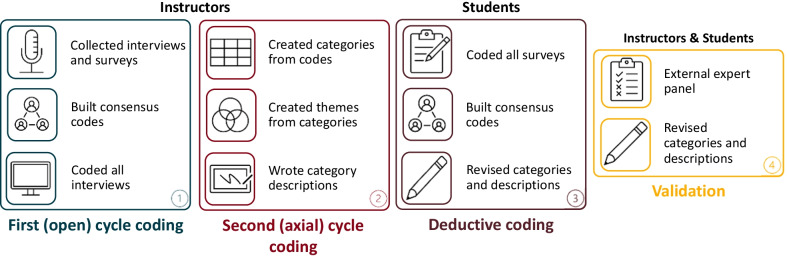
Qualitative coding and validation approach. We completed first cycle coding for instructor interviews followed by second cycle coding where we generated categories and themes from initial codes. Following instructor data analysis, we coded student survey data using categories generated previously by the instructor data. Following instructor and student analysis, an external expert panel was used to validate categories and descriptions. Blue represents first or open cycle coding, red represents second cycle coding, dark red represents deductive coding with student data, and yellow represents study validation

Grounded theory techniques (Charmaz, 2006; Corbin & Strauss, 2014) in qualitative analysis employ a rigorous, iterative process of examining the properties and dimensions of data to create a holistic understanding of a process or phenomenon (Charmaz, 2006; Corbin & Strauss, 1990; Miles et al., 2018). Drawing from grounded theory methods, we used two cycle qualitative analysis (Miles et al., 2018) to explore instructor interview transcripts in a section-by-section fashion (Fig. 3).

#### First (open) cycle coding

First-cycle qualitative coding allows researchers to gain a comprehensive and integrated view of a data set (Miles et al., 2018). It is intentionally cyclical, such that a code generated during the first cycle is not meant to be a static assessment. Rather, fluidity is essential. As we engaged in first cycle analysis, we used open coding to look holistically across all the data and identified repeating indicators of instructors’ perceptions of teaching during the COVID-19 pandemic. Strauss and Corbin (1998) described open coding as a process of examining properties and dimensions that exist within the data, allowing the researcher to identify unique and discrete aspects (Miles et al., 2018). To do this, we looked across all instructor transcripts and began assigning codes that indicated how instructors perceived supports and barriers, while they conducted ERT. Table 3 demonstrates what first cycle, open coding looked like for one of the two researchers (CD and EM).

**Table 3:** First (open) cycle coding

ID	Dialogue	CD initial code
Interviewer	*What would you say was the biggest concern for the transition? I know you said the communication with students was bad*	
Samuel	*For what I saw on the students’ side, access to technology was sometimes difficult. I didn’t have too many examples, but a recent thing is one student, their parents have both lost their job, and so, the student has to start a job on their own, working as a fruit picker in the farm, and not being able to have the availability for exam minutes, before. So, asking for an exception to take the exam*	**Barrier**: lack of student access to technology; **Barrier**: Student obligations and pressures

Prior to moving into the second cycle of analysis, we engaged in consensus-building with five researchers (CD, EM, PK, WA, and HB). Consensus building in qualitative analysis is a critical measure of ensuring validity and trustworthiness (Corbin & Strauss, 2014). To address inter-coder consistency, we independently coded approximately 10% of all transcripts, and then we used discussion-based consensus building to address discrepancies in codes. Saldaña (2015) describes this process as *interpretive convergence*, specifically useful in qualitative analysis, where dynamic interpretations of data are paramount, as opposed to seeking statistical significance in quantitative methodologies. In our efforts to converge toward common codes, we discussed both our individual open codes as well as our analytic memos. Analytic memos in qualitative analysis serve as a researcher’s dialogue, both with themselves and each other, about what the codes mean to them as they are coding (Charmaz, 2006; Corbin & Strauss, 2014; Miles et al., 2018; Stake, 2005). Table 4 depicts open codes and analytic memos during one of our consensus coding sessions. It shows five of the author’s initial codes, generated individually, and the consensus code generated as a group after discussion.

**Table 4:** Consensus coding

**Dialogue**					
*So one of the challenges there is to prevent students from communicating, but we couldn’t do it with a large class just like we had with Bio 2. One section was over 200 students. So what I had to do was use a trick. ’Cause we were using breakout rooms. Maybe, I’ll come back to that in a different question. But to finish off your question here, I’ve tried at Stanislaus to have 150 students on camera with their microphones, and it worked. Although, three students did drop and have to reconnect. So I found that useful to have pictures of that—all the students taking the exam using either their screen camera, or if they didn’t have a working computer camera, I made them put their phone next to them and theirselves so I could see what their eyes were looking at. –- Kailish*					

Initial codes					
CD	EM	PK	WA	HB	Consensus
Challenge to academic integrity; made students use camera	Student academic integrity; preventing cheating	Student videos are possible and preferred; instructor monitors students during in-lecture, timed exams to reduce academic misconduct	Previous experience; prefers students to have webcams on	Couldn’t prevent students from communicating	**Barrier**: Monitoring students during synchronous exams to prevent cheating

Analytic Memos					
CD	EM	PK	WA	HB	
		Student academic conduct during exams seems like a key challenge for instructors teaching with ERT	Instructor has experience with having a large number of participants in a video call on webcams	Others have mentioned academic honesty/integrity in this section too	

#### Second (axial) cycle analysis

The aim of second cycle analysis is to find linkages between the discrete parts that were earlier identified in open coding (Miles et al., 2018), to find “broader categories, themes, theories, and/or assertions” (p. 234). Essentially, this involves looking for similarities and differences across the previously identified properties and dimensions of the data set (Strauss & Corbin, 1998). We engaged in this process of creating relational categories through axial coding. Tables 5 and 6 depict our initial step in second-cycle analysis, wherein two researchers (CD and EM) took a second, critical look at the data set and compared similarities and differences in codes. Once they reached consensus, then they began organizing codes into categories based on their coding notes, consensus conversations, and analytic memos. Table 9 showcases those categories.

**Table 5:** Second (axial) cycle consensus coding with support example

ID	Dialogue	CD initial code	EM initial code	Consensus code
Interviewer	*So let’s start with some questions about your experience after the transition to emergency remote teaching during the COVID-19 pandemic. So what did you find helped or supported your teaching?*			
Katherine	*Well, I guess getting the Zoom installed pretty quickly because I didn’t have that installed or an account for that. But also, I guess what helped was, I don’t know, I guess having the tools necessary. Because I know some TAs didn’t have a touchscreen laptop or whatever, so it made it more difficult to teach. I mean, I had my tablet that I could use, so I feel like that made it really helpful. Because then, I could just—I also recorded the lecture so I can share it with them*	**Support:** Quick access to technology	**Support**: Unsolicited timely consistent technical support and technical resources from IT	**Support:** Quick access to Zoom; Support: Access to tools for remote instruction

Note: There were no analytic memos for this example

**Table 6:** Second (axial) cycle consensus coding with barrier example

ID	Dialogue	CD initial code	EM initial code	Consensus code
Interviewer	*What would you say was the biggest concern for the transition? I know you said the communication with students was bad*			
Samuel	*For what I saw on the students’ side, access to technology was sometimes difficult. I didn’t have too many examples, but a recent thing is one student, their parents have both lost their job, and so, the student has to start a job on their own, working as a fruit picker in the farm, and not being able to have the availability for exam minutes, before. So, asking for an exception to take the exam*	**Barrier**: Lack of student access to technology; **Barrier**: Student obligations and pressures	**Barrier:** Student access to technology; **Barrier**: Student obligations and pressures	**Barrier**: Lack of student access to technology; **Barrier**: Student obligations and pressures

Note: There were no analytic memos for this example

Four researchers (CD, EM, PK, and HB) then worked together to make descriptions for supports and barriers categories. The following categories and descriptions emerged from the data (Additional file 1: Table S1). Support categories included: prior experience, timing, technology for remote teaching, community, help with technology, socio-emotional factors, teacher beliefs, working from home, help with teaching, course attributes, student comfort interacting online, and reducing cognitive load. Barrier categories included: communication difficulties, time management, instructor teaching inexperience, instructor technology issues, teaching and learning resources, student integrity, administrative issues, student presence and participation, student emotion and comfort, student technical issues, assessment difficulties, instructor emotion, responsibility and workload, and instructional space. We engaged in several measures to examine if and how we were reaching theoretical saturation in our analysis, including multiple feedback sessions with an external, expert review panel, as well as with researchers of this same lab group that were not immersed in the data set.

#### Student deductive coding

A deductive coding approach was used to identify the various student support and barrier categories generated from coding the instructor interviews (Table 2). First, two researchers (PK and MCK) independently coded the student responses using the 12 support categories (Table 9, Additional file 1: Table S7) or 14 barrier categories (Tale 10, Additional file 1: Table S8) to get initial codes (Table 7). Each student response was one to three sentences in length and could contain more than one category.

**Table 7:** Individual coding with support example

ID	Student response	MCK initial code	PK initial code
Skyler	*Online lecture recordings allowed me to revisit past material to ensure I was understanding the taught concepts*	Preexisting course structure	Classroom technology

Note: There were no analytic memos for this example

Next, PK and MCK met to discuss their categories until reaching 100% consensus (Table 8). When coding student responses, authors noticed that the working descriptions of some categories would only fit the instructor perspective and needed to be redefined to be used for student coding. Authors then met to discuss which descriptions should be changed and rewrote them so that the codes could be used for both students and instructors alike.

**Table 8:** Consensus coding with barrier example

ID	Response	PK	MCK	Consensus
Jessie	*Even before quarantine, I would have trouble doing the labs but I would go to office hours and then chem center, but now it is more difficult to do that because I am more of a hands-on learner*	Teaching Resources	Student Technical Issues	**Barrier:** Teaching and Learning Resource

**Analytic Memos**				
**PK**	**MCK**			
Need to modify “teaching resources” to “teaching and learning resources” as this describes not having access to learning resources	Trouble with something before COVID, but can’t access the resources that they used to. Maybe technical issues, lack of face-to-face support and lack of student resources			

#### Validation

Following student coding using the categories and agreed upon descriptions, we brought the categories, themes, and descriptions of categories to an expert feedback panel of five. The expert feedback panel was made-up of STEM educators (both biology and chemistry), discipline-based education researchers (DBER), and learning scientists at a research-intensive institution unrelated to the one in this study. This expertise allowed the panel to provide valuable feedback on category descriptions.

The formatting for the feedback was organized in two parts. For the first part, the first author (CD) presented the support instructor and student themes, categories, and their descriptions along with examples (Additional file 1: Table S1). Expert feedback panelists were then allowed to ask questions prior to a short content validation. This content validation consisted of splitting the panelists into two groups and providing each group with the same three *support* quotes representative of categories in each of the three themes (Additional file 1: Tables S3 and S4). The panelists were given all the support categories and descriptions and were asked to match the quote with a category and provide justification via notes. In addition, authors CD, PK and JA were present in both groups to take notes on feedback. After each group had finished choosing categories for the quotes, everyone met back in one group and the answers were discussed. Comments and feedback are reported in Additional file 1: Tables S3 and S4. Following this discussion, the *barriers*, their descriptions, and themes were then presented by CD to the expert feedback panel (Additional file 1: Table S2). The same two groups were then created, and panelists were again given the list of barriers and their descriptions and asked to decide which category fit each quote best. After each group had finished, all comments and questions were recorded (Additional file 1: Tables S5 and S6). Again, CD, PK, and JA were present in both groups to take notes on feedback.

Following the expert feedback panel, all coders met and discussed the feedback. This discussion allowed changes from version 2 to version 3 for categories and descriptions (Additional file 1: Tables S7 and S8). These are the final categories and descriptions presented in this manuscript.

## Results

The findings presented here are separated by instructor and student and then into the two primary areas of study, supports and barriers. For the instructors, we specifically discussed categories and themes as these data were inductively derived from our instructor interviews. We then assessed student data through deductive coding by utilizing our categories that were inductively derived from instructors (Fig. 3).

We identified 134 unique support codes which led to the development of 12 categories, and, ultimately, three support themes: 1) tools and support for class content, 2) mental and emotional support, and 3) preexisting supports. A total of 203 unique barrier codes were identified, which led to the development of 14 categories from which the following three barrier themes emerged: 1) structures getting in the way, 2) spending more time and effort, and 3) affective issues*.* For definitions of all categories, please refer to Additional file 1: Tables S7 and S8.

### Instructor supports

Instructors described a variety of supports when discussing their switch to ERT. These supports ranged from existing structures that made remote teaching easier, to emotional support from colleagues. Here, we describe how we went from transcript to themes focusing on the parts of themes that help define them (Table 9).

#### Instructor open coding for supports

By starting with open coding, we were able to inductively determine which codes were present in all transcripts. This process allowed us to develop codes that may not have been present in an already formalized coding tool.

An example support is given below:

*Yeah. So I needed some technical help, just advice about online exams and best practices for doing synchronized learning. But then, the DBER [journal] Club and the scientific institutes, those were also just moral support types of help. Just knowing that it was new for everyone, we’re all struggling, and hearing from other folks about particular types of issues to figure out if what I was experiencing was similar or different and, yeah, just getting ideas about how to handle different situations. ---Laney*

When Laney was asked about what she felt supported her teaching or learning, she brings up the DBER [Journal] Club, which stands for discipline-based education research. This is a group that meets weekly throughout the year to discuss science education research and different science education research articles. It is made up of a large mix of STEM people, ranging from full professors to undergraduates that spans all the disciplines. Participants in this group have a large variety of DBER experience allowing for a generally well-balanced discussion of topics. In addition, particularly during the transition to ERT, this group took several different weeks to discuss tactics and strategies that people were either currently using, planning on using, or heard about regarding pandemic teaching. This weekly meeting is also set up so that during the first 15–30 min of each 1.5-h meeting, there is time for informal chatting. Many members use this time to commiserate with each other by sharing frustration and/or offering moral support to each other. It is also noteworthy that several members of this group held administrative roles in the university that allowed them to share timely and relevant information with the other group members. For example, one member was involved in information technology (IT) research infrastructure, so they were able to support instructors with changes to classroom technology during ERT.

During the open coding process of Laney’s quote, CD identified supports that were both technical and moral, while EM identified a support of hearing how others were handling the situation. During the consensus process, discussion between the consensus coders focused on whether to differentiate between technical and moral support, how specific to be with respect to technical support, and whether it was the people or the discussions that were the actual support. This discussion resulted in two codes:

1. Conversations with colleagues about potential ideas and practices
2. Casual conversations with colleagues for moral support

#### Instructor categories for supports

After reaching consensus on all the transcripts, the researchers individually collected the codes into categories, then met to resolve any differences. This process led to the creation of 12 categories, shown in Table 9 and Additional file 1: Table S7. These categories covered topics, such as access to technology, help with remote teaching pedagogy, access to a supportive community, the comfort of working from home, and the timing of the switch to ERT. As an example of the support categories, consider the two quotes below after instructors were asked what supported their teaching:

*I’d say getting the Canvas website very organized has supported the class content delivery. [Claire] ended up looking at my class when I was weaving up the website when all this had to happen—when I was putting this online. So having somebody who had developed detailed Canvas modules before—just gave me a few tips—was really useful right then. ---Constance*

*I felt like, specifically at UC Merced, there was this general community, this sense of community among all of the colleagues and everybody banding together and making it—making the whole learning environment as good as it could be. That’s what it felt like. I felt very fortunate to be at UC Merced working with all these really cool people who are doing the best that they can to make sure that everything’s hunky dory. ---Josephine*

On the surface both quotes seem similar, in that both Constance and Josephine identified people they could talk to as a support. However, Constance’s quote points to the support of having someone available to answer specific, course-related questions about best practices, whereas Josephine’s quote is more about the camaraderie of people in similar situations and knowing that others are persevering under difficult circumstances. It is of note that UC Merced is a newer university (established in 2005) and as such is still small by many research institution standards (about 9,000 undergraduate and graduate students). This creates an environment, where instructors ‘wear many hats.’ That is, not only do the instructors at UC Merced run top-level research laboratories and teach, but their service is exponentially higher than other UCs. Therefore, many instructors who were more experienced in certain areas, such as setting up clear and concise modules on their learning management system (LMS), showed their less experienced colleagues how they did it during ERT.

As a result of the differences between quotes, Constance’s quote was coded as ‘help from more experienced colleagues,’ whereas Josephine’s quote was coded as ‘community of colleagues.’ These differences also led to the quotes being placed in different categories. As Constance’s quote was focused primarily on pedagogical help, it was placed in the ‘help with teaching’ category, whereas Josephine’s quote, which focused much more on the emotional support of others, was placed in the ‘community’ category.

#### Instructor themes for supports

Once the codes were categorized, the researchers individually organized them into themes, and then met to resolve any differences. This process led to the creation of three broad themes of: 1) tools and support for class content, 2) mental/emotional support, and 3) preexisting supports, as shown in Table 9. Here, we look at a quote from Chase describing what he felt supported his teaching:

*So in the classroom, we’ve been using Canvas and Zoom a lot. We’ve been doing breakout sessions, making sure to—mostly with follow-up e-mails a lot, things like that, personally wired headsets, stuff like that, as far as hardware and such like that. ---Chase*

Chase’s quote was representative of the supports that fell into the ‘tools and support for class content’ theme. These were supports those instructors identified as helping them deliver or develop material in a remote environment. Other examples of this theme described by instructors were workshops from UC Merced’s CETL, online groups that instructors could turn to with pedagogical questions, and the ability to host videos through sites like YouTube. It is perhaps expected that when instructors are asked about what supported their teaching during ERT that ‘tools and support for class content’ would be a common theme. This manuscript aims to provide specific examples through categories that could be used for future ERT situations (Table 9).

**Table 9:** Themes, categories, and example open codes for supports

Example Open Codes	Category	Theme
Access to tools for remote instruction Breakout rooms in Zoom Good internet for instructor	Classroom technology for remote teaching	Tools and support for class content
[Mitch]’s remote teaching document Workshops and training from CETL Support from IT with Zoom	Help with technology	
Having a hard-working TA Help from more experienced colleagues Staff support for developing lab material	Help with teaching	
Peer supporter Informal conversations with colleagues Community of colleagues	Community	Mental/emotional support
Students understanding/supportive of instructor Flexible mindset Supportive administrative attitude	Socio-emotional factors	
Personal confidence Self-motivation Autonomy was empowering	Teacher beliefs	
Quiet place to work Spouse and pets Able to take a quick nap	Work from home	
IOR created highly structured transition Small changes to accommodate ERT rather than large changes SNS staff made things happen faster	Reducing cognitive load	
Prior knowledge with iPad Prior training about using Canvas well Prior videoconferencing technology knowledge and experience	Prior experience	Preexisting supports
Early warning about transition Meeting as a class prior to ERT Timing of switch to ERT	Timing	
Flipped class structure made it easy to switch to remote Having a small class Class structure was easy to transition to remote environment	Course attributes	
Student comfort with online experience Student familiarity with streaming platform Students seemed more comfortable asking questions	Student comfort interacting online	

Next, Yuan discussed what supported her teaching:

*Well, for me, personally, I think the students have been very good, very supportive. Even though I don’t see them anymore, they still have a very good attitude and participate in lectures and worked very hard, take it pretty seriously. So that’s emotionally or psychologically, that’s a very positive factor. ---Yuan*

Yuan is describing how her students’ attitudes were a strong support for her. She discusses how their hard work and good attitudes provided positive emotional support for her. Her quote was representative of the supports that fell into the ‘mental/emotional support’ theme, which are supports that help instructors handle the additional stress and emotional toll of working in a remote environment under unfamiliar conditions. Other examples of this theme were having a supportive partner at home, enjoying the challenge of trying something new, and having a well-developed transition plan to minimize confusion.

When we asked Danielle about her supports during the transition to ERT, she specifically mentioned her prior experience with the technology. She said:

*One is because of another project I worked on, I was familiar with making videos and editing videos. So the technology was familiar to me. I was already somewhat familiar with Zoom. I’ve already had been doing a fair amount of using CatCourses and Top Hat and web-based tools for teaching alongside my face-to-face. So it wasn’t like I went from totally in class to totally not. I think that helped. ---Danielle*

Danielle described how she did not struggle as much with her transition to ERT, because she had already needed to learn how to do many of the things her other colleagues were struggling with before ERT. Her previous experience on a project taught her how to make and edit videos as well as how to use Zoom. In addition, she mentioned how she already utilized CatCourses (i.e., Canvas, an LMS, for UC Merced) and Top Hat tools in her course, allowing for a more seamless transition from in person to ERT, because she did not have to reformat anything on her LMS.

Danielle’s quote was representative of the supports that fell into the ‘preexisting supports’ theme. These were supports that were already in place that inadvertently made the transition to ERT easier for the instructors. Other examples were instructors who had prior experience with online teaching, student familiarity with streaming services, and courses previously delivered using a flipped modality.

### Instructor barriers

Following supports, we found a larger variety of barriers described by instructors discussing their transition to ERT. These barriers covered issues ranging from instructor access to technology to concerns about academic integrity. Here again, we describe how we went from transcript to themes focusing on the parts of themes that helped define them (Table 10).

**Table 10:** Themes, categories, and sample codes for barriers

Example Open Code	Category	Theme
Harder to engage with students Difficult to encourage peer–peer interactions Hard to communicate math via Zoom	Communication difficulties	Structures getting in the way
Delays getting Zoom licenses Instructor access to technology Low instructor internet bandwidth	Instructor technology issues	
IT interference with video creation Institutional pressure to be primary communicator Lack of clear shutdown timeline	Administrative issues	
Student bandwidth limitations Lack of student access to technology Lack of face-to-face technical support for students	Student technical issues	
Having TA grade in Canvas Assessing if students are watching videos Incorporating formative assessment	Assessment difficulties	
Background noise during class time Cooperative and group learning didn’t feel feasible Harder to be aware of student issues and motivation	Instructional space	
Covering material more slowly Hard to cover all material by remote instruction Time necessary to develop/modify materials for online labs	Time management	Spending more time and effort
Inability to monitor student attention distracted instructor Finding the right balance of pace and flexibility Figuring out how to transition the labs to online format	Instructor teaching inexperience	
Institutional training didn’t match instructor’s need Lack of staff help to prepare for ERT prior to ERT Difficulty finding necessary resources	Teaching and learning resources	
Managing TAs was challenging Responsibility for adjunct instructors Needing to be in total control of course	Responsibility and workload	
Decreased student accountability Students were less interactive Decrease in office hour attendance	Student presence and participation	
Concerns about academic integrity Hard to prevent cheating in online exams Not being too flexible to avoid being taken advantage of	Student integrity	Affective issues
Concerns about student mental health Not knowing the student work environment conditions Student name visibility	Student emotion and comfort	
Frustration with student preparation for lecture Zoom fatigue Difficulties managing work life balance	Instructor emotion	

#### Instructor open coding for barriers

An example barrier from Diane is given below. After she was asked about the decline in her student participation, she mentioned in an earlier part of her interview:

*No, it was a slow decline toward the end of the semester. And you usually get that in classes, anyways, but I was noticing it a whole lot more. Some students just become overwhelmed and slowly stop turning in assignments, but this seemed a little more pronounced than what I’ve usually seen. ---Diane*

During the open coding process, CD identified a decrease in student participation as a barrier, while EM identified a decrease in quality of student work as a barrier. During the consensus process, discussion between the consensus coders focused on gauging participation and measuring quality, which resulted in barrier code of ‘decrease in student assignment submissions.’ After consensus coding all the barriers, a total of 203 unique barriers were identified.

#### Instructor categories for barriers

While there were many more barriers than supports coded, the barriers collapsed into a similar number of categories (14 barrier categories, compared to 12 support categories), as shown in Table 10 and Additional file 1: Table S8. These categories covered topics, such as issues with instructors and students using technology, increased responsibility and workload, decreased student motivation, and concerns about student integrity. As an example of how these barrier categories came about, consider the two quotes from Arturo and Roberto below, where both instructors were asked what barriers did not support them during their transition to ERT:

*With a pre-recorded video, that’s impossible. Then, students won’t even watch it. I was convinced I shouldn’t do asynchronous. Then, through the experience, I discovered that conviction was reinforced, because the students told me. I asked them multiple times, and they told me, “This is much better precisely because we can stop you and just ask you to explain the thing, again.” ---Arturo*

*It’s up to them to use their time the way they want to do it, but what we’re finding is it’s hard to project with the statistics whether they’re actually watching the videos or skimming through the videos or how many times they’re watching the videos. That’s hard data to obtain. The only way to get at it is to maybe get an assignment back from them—a lab or a quiz or whatever. You covered some of the stuff on the videos and kind of points out, “These are some of your flaws that you’re not really looking at the videos.” ---Roberto*

Like the support quotes discussed above, both Arturo and Roberto identified barriers that, on the surface, seemed similar in that they both present difficulties with video lectures. However, Arturo’s quote really pointed to the difficulty of getting immediate feedback from students about specific topics, whereas Roberto’s quote was more about collecting data about whether students are really watching the videos. As a result of these differences, Arturo’s quote was coded as ‘asynchronous teaching was not possible due to students not being able to provide timely feedback about content understanding’, while Roberto’s quote was coded as ‘assessing if students are watching videos.’ In addition, these differences led to the codes being placed in different categories. Because Arturo’s quote was about the potential difficulty of engaging and interacting with students via an asynchronous modality, his code ended up in the ‘instructional space’ category and Roberto’s quote, which focused much more on difficulties measuring student interaction, ended up in the ‘assessment difficulties’ category.

It is perhaps important to note that both Arturo and Roberto held different instructor positions in different STEM departments. Arturo goes on to explain that his use of synchronous teaching increased student participation, whereas Roberto struggled to get his students to participate throughout the entire experience. Due to Roberto’s position as a non-tenure track lecturer, perhaps he did not feel comfortable going against the departments expectations to provide asynchronous material to the students, whereas Arturo, a tenure track faculty member, might have felt more autonomy to make changes in his classroom.

#### Instructor themes for barriers

Once the codes were categorized, we individually organized them into themes, and then met to resolve any differences. This process led to the creation of three broad themes of: 1) structures getting in the way, 2) spending more time and effort, and 3) affective issues, shown in Table 10. Exemplar quotes for these three themes are shown below.

*And I think, sometimes, it assumes that what happens in those meetings is being shared with other faculty, and that’s not always the case. And so it would have been nice to hear some of that earlier on. And I think going forward that that should just be made explicitly clear that more information is better, I guess, especially when we’re all separated from each other. So that would have been a nice support to have. ---Laney*

Laney’s quote was representative of the barriers that fell into the ‘structures getting in the way’ theme. These were barriers that instructors identified as preventing them from quickly and efficiently transitioning to ERT. Laney described how some faculty participated in meetings at the departmental, school, or institutional levels and assumed that leaders of those groups or committees were sharing information pertinent to transition through ERT beyond those meetings. However, often that was not the case, and faculty not in attendance at those meetings would not receive crucial information that might impact their teaching efforts. Other examples of this theme were poor bandwidth for instructors, and students, lack of access to a quiet workspace, and difficulty writing math equations in Zoom.

*Then I figured out, oh, they can see everybody in the entire class. Then it wouldn’t make it a group discussion. Then I put students in groups where each group is all the students in the discussion session, and the time it took to put that together just sucked. So much of my time got sucked up with this bureaucratic, digital—I had to click buttons. I had to move students. I had to cross-reference things. ---Claire*

Claire’s quote was representative of the barriers that fell into the ‘spending more time and effort’ theme. These were barriers associated with instructors working less efficiently. Claire specifically described spending a lot of time learning and setting up her course digitally. Prior to Zoom updating their breakout room capabilities, instructors had to manually enter students into groups if their student numbers were over 200. Claire discussed how this digital maneuvering really took up a lot of time in class. Other examples of this theme were having to take time to teach other instructors how to teach remotely, working harder to have students engage with the material, and the inefficiency of communicating via Zoom.

*I know, and also, my big concerns were for the mental health of my students because they all—my class is really unique, they all live together… and they are all each other’s best friends, and then they just got ripped apart from each other. At the beginning of the week everything was fine, and then that Friday everybody was home and they weren’t seeing each other for the rest of the semester. And I know that that was tough on them. And so I was concerned for everybody’s wellbeing. ---Kiera*

Kiera’s quote was representative of the barriers that fell into the ‘affective issues’ theme. Kiera was the instructor for a unique course, where the students that attended were students that lived in the same dorm. These living and learning communities (LLCs) united students who had similar interests and educational goals with academic, social, and personal resources across the campus community. The LLCs aimed to generate a greater sense of belonging and building community. Therefore, as described by Kiera, it was particularly difficult for a course that was aimed at fostering these ideals to suddenly go virtual during ERT.

The barrier theme ‘affective issues’ was associated with instructors dealing with negative emotions, either their own or from their students. Other examples of this theme were worrying about student academic integrity, witnessing abusive home environments, and feeling guilty about not being an expert in remote teaching.

### Student supports

**Fig. 4:**
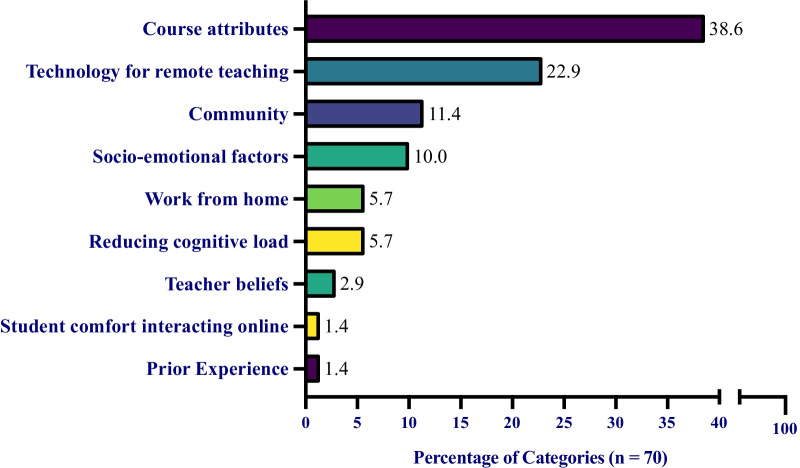
Students’ perceived supports during their transition to ERT. The percentage of support categories from students. Each value is the total number of each category divided by the total number of all categories, 70. For example, ‘course attributes’ was mentioned 27 times and 27/70 = 38.6%. Note: no student used a single code more than once

Students described nine supports when discussing what helped their learning through the transition to ERT, ranging from course attributes to prior experiences that students had before the start of ERT (Table 9). However, ‘course attribute’s (38.6%), ‘technology for remote teaching’ (22.9%), and ‘community’ (11.4%) were the three most frequent categories described by students as supporting their learning during ERT (Fig. 4). Access to technology and reliable internet is critical for all students during ERT; however, this access was not guaranteed for our students. The fact that ‘technology for remote teaching’ was one of the top supports chosen by students speaks to UC Merced’s ability to provide the necessary technology for students. Not only did UC Merced provide the necessary technology, but they were also able to effectively let students know that it was available and made it convenient enough for students to find it helpful. Previous semesters had technology distribution available, but there were limitations, such as only allowing loans for a short period of time (i.e., up to 1 month) and not advertising the technology resource well enough for students who needed it.

Here, we describe how we went from student survey responses to categories for Claire’s class. For example, in response to the written question asking what supported their learning, one student wrote that:

Homework assignments and discussion sections with my TA were extremely helpful, and the lab sections also helped. –-Sammi

AT UC Merced, many of the large enrollment lectures are associated with a required smaller discussion and lab section. These discussions and labs are made up of no more than 24 students per section and are generally taught by graduate TAs. Homework assignments, discussions sections, and lab sections were all attributes of the course that had existed prior to the transition to ERT. Therefore, Sammi’s quote was placed into the category ‘course attributes,’ since the student discussed components of the course (e.g., homework assignments) that helped them ease into the transition to ERT. This was the most frequent support category described by students.

Two other students, Ash and Jax, wrote that:

*I think the instructional videos provided helped us greatly in learning the content of the course. Zoom calls helped us catch up and know his outlook on the plan for the coming week of the course. ---Ash*

*Zoom discussions, zoom lectures, recorded lectures, online tutoring services outside of school –-Jax*

Ash stated that features of the Zoom platform aided their learning, which lead to their response being put in the category’ classroom technology.’ Ash is in instructor Haik’s class. This instructor created office hours during class time so that he knew his students could attend them. He called these Zoom lectures but treated them like office hours. In addition, student Jax listed Zoom lectures, discussions, and other online sources as resources that helped their learning, which lead to this response being placed in the category ‘classroom technology.’

Finally, another student wrote that:

*Having online lectures actually felt like I was more connected to the class rather than in person, so that really helped me out. –-Ekene*

This quote was placed in the category ‘community’ because of the connection to the class that aided the student and helped them sustain their learning. This student quote was not surprising to us. During the transition to ERT, UC Merced’s CETL provided training for faculty to help them build community and sense of belonging in their classrooms. They recommended regular “check-ins” at the beginning of class to check on students’ well-being and create space and time for informal interactions during synchronous lectures.

### Student barrier categories

**Fig. 5:**
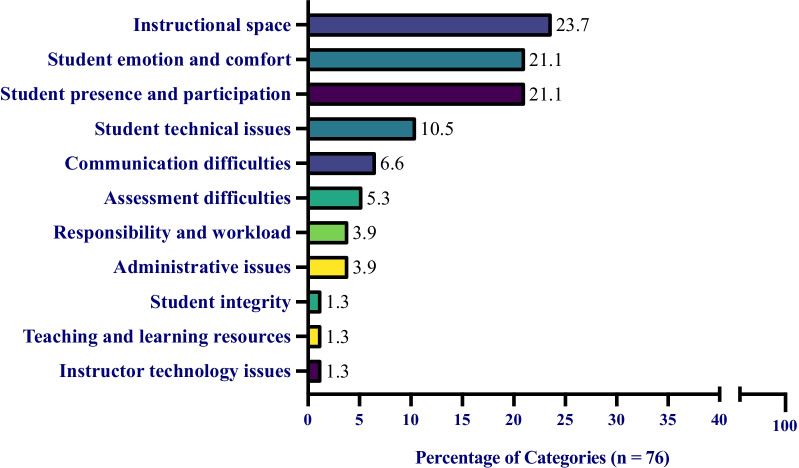
Student perceived barriers during their transition to ERT. The percentage of barriers from students. Each value is the total number of each category divided by the total number of all categories, 76. For example, ‘instructional space’ was mentioned 18 times and 18/76 = 23.7%. Note: no student used a single code more than once

Students described ten barriers when discussing what challenged their learning during ERT. These barriers ranged from concepts, such as instructor technology issues to instructional spaces (Table 10). However, ‘instructional space’ (23.7%), ‘student emotion and comfort’ (21.1%), and ‘student presence and participation’ (21.1%) were the most frequent categories students found challenging their learning during ERT (Fig. 5). Here, we described how we went from student survey responses to categories for students from Constance’s class. For example, two students wrote that:

*I felt there wasn’t much learned as if I were in person. –-Nasim*

*Not being able to study in groups or ask my friend to help explain my questions. Also, there are fewer physical demonstrations in classes and I tend to get more distracted at home –-Ollie*

Nasim described feeling as if they were not learning as much remotely as in-person. In addition, Ollie described how the remote learning environment was difficult and distracting compared to the in-person learning environment. Therefore, both quotes were put in the category ‘instructional space.’

In addition, two students wrote that:

*It was hard to focus at home because there were a couple of distractions. Also I kind of lost some motivation through online learning. –-Xia*

*Productivity was a challenge of mine. I live in a household with younger siblings who are not only rowdy but also need help with their online classes as well. –-Rio*

There were many different reasons that might have prevented students from attending or participating in course activities or office hours. These factors can range from different emotional states to familial responsibilities and could have had a negative impact on students’ learning. Because Xia and Rio described facing some of these difficulties causing them to be unproductive, they were placed in the category ‘student presence and participation.’ UC Merced has a large portion of first-generation and low-income students who may still be expected by their family to contribute to helping younger siblings when at home and/or working to support their family (University of California, 2020).

Finally, one student wrote that:

*When we transitioned to [video only] lectures. I felt cut off and sort of isolated from the class. It was starting to feel pretty lonely and discouraging. –-Mitra*

Because Mitra wrote that this isolation was a barrier to their learning, and it resulted in them feeling lonely and discouraged, it was listed as ‘student emotion and comfort.’ We placed it in that category, because we felt that it could be included as a concern for student emotional well-being. College students in general suffer from high rates of poor mental health (Lipson et al., 2019). The pandemic exacerbated students known mental health risk factors while simultaneously putting students’ academic outcomes in jeopardy. It is also known that the demand for mental health services exceeded the resources on most campuses, and UC Merced is no exception (LeViness et al., 2019). Access to mental health is particularly difficult in the Central Valley of California (where UC Merced is located), especially for people from lower socioeconomic backgrounds (Lama et al., 2018). It is, therefore, critical that we pay close attention to students, such as Mitra, who expressed feelings of loneliness and discouragement during ERT.

### Instructor and student comparisons

To further investigate the student experience, we decided to compare the student’s responses to their instructors’ responses. We wanted to know if the students that we surveyed had a similar experience in their transition to ERT as their instructors. It was fortunate that we had all four instructors of the students surveyed as part of our instructor data. All the student data came from students with at least one course taught by an instructor that also participated in our study, and we wanted to compare the supports and barriers identified by the students with these four instructors. When assessing the instructor and student category data, we found that there were more barriers described by students than supports, like the instructors’ results. The most frequent support categories applicable to students were ‘course attributes’, ‘technology for remote teaching’, and ‘community’. In comparison, the instructors of those students’ most frequent support categories were ‘community’, ‘help with teaching,’ and ‘help with technology.’ We found that both instructors and students overlapped with mentioning the ‘community’ category defined as a “personal or professional network to sustain teaching, learning, or yourself.” It is understandable that both students and instructors alike felt ‘community’ was one of their biggest supports. For example, below one instructor and one student state:

*So by and large, colleagues, and a broader, especially for the courses I teach, a pretty strong community, a professional community outside of the campus. So other specialists in my area that are also engaged in teaching the same kinds of classes ---Haik (Instructor)*

*I like how professor [Instructor Haik] set up a hybrid online course. This allowed me to view the lecture videos on my own time and I still had the opportunity to ask questions and listen to the questions of my peers during zoom lectures. ---Mitra (Student)*

If we look at Haik, the instructor, and one of his students, Mitra, we can see how they both described their perspective colleagues as part of their supportive community. Instructor Haik discussed how his community of support was other specialists in his area of research and Mitra discussed how their peers during synchronous teaching provided support to them.

The categories ‘instructor teaching inexperience’ and ‘time management’ were some of the most common categories from instructors as barriers to their transition to ERT. However, neither of these categories were mentioned by students, showing that even though there may be overlapping supports between students and instructors, the barriers seem to differ more. One common barrier cited by instructors and students was the category ‘instructional space,’ which we have described as “difficulty implementing or participating in teaching and learning activities.”

*With breakout rooms, students weren’t really—they needed to be introduced and encouraged, kind of trained, on use. Show your video. Talk to each other. Initiate the conversation. I also found it really difficult that if I was in a breakout room, nobody could contact me. If my TA was like, hey, you need to come back, they couldn’t reach me. ---Claire (Instructor)*

*The difficulty of focusing given the format, and the impersonal-ness of lectures, as well as the difficulty of following lectures when the pace is not as connected with the students’ ability to keep up. ---Sammi (Student)*

The instructor Claire and one of her students Sammi both described how ‘instructional space’ inhibited their teaching or learning. Claire described how she found it difficult to get students to work in groups and find the students and groups that needed her help. Sammi felt it was difficult to focus with the format that Claire was using, as well as they felt it was impersonal and did not connect to their pace of learning. It seems that Claire and Sammi both struggled with Zoom, especially the breakout room feature, as the ERT teaching platform.

Given our themes and categories above, as well as our interest in why the *teaching*, *social*, and *cognitive presence* might be different during ERT, we wanted to contextualize the support and barrier categories within the COI framework. To do this, we will focus on understanding the positive and negative impacts on *teaching presence*, *social presence*, and *cognitive presence* during the transition to ERT (Fig. 2).

### Impact on *teaching presence*

*Teaching presence* is defined as “the design, facilitation, and direction of cognitive and social processes for the purpose of realizing personally meaningful and educational worthwhile learning outcomes” (Anderson et al., 2001). One of the themes we considered a support for *teaching presence* was ‘tools and support for class content.’ Having communication tools and the right technology was critical for both students’ and instructor’s success during the transition to ERT. For example, several instructors mentioned how their early access to technology allowed them to quickly transition from in-person to remote. This quick transition helped them maintain their *teaching presence*. In contrast, the instructors that had difficulties with technology, such as delays getting Zoom licenses or poor internet connection, fell under the theme ‘structures getting in the way.’ This theme of ‘structures getting in the way’ directly hindered instructors’ ability to provide and maintain a consistent *teaching presence*, thereby hindering student’s ability to experience a strong *teaching presence*.

An example of a support category we considered an influence on *teaching presence* was the category ‘prior experience.’ We defined prior experience as “knowledge, skills, and experiences before the start of ERT.” For example, instructor Diane said: “We did an interview with a Skype scientist, and Zoom worked really well for that, because I was already using Skype, so I just transitioned to that platform,” which indicated her prior knowledge with Skype was a contributing factor to her ability to quickly transition to ERT, and it allowed her to maintain her *teaching presence*, largely unchanged, for that aspect of the course. In addition, Diane’s prior knowledge was a support in the switch to ERT, because it allowed her to maintain meaningful learning outcomes for her students.

In contrast, an example of a barrier category we considered a negative influence on *teaching presence* was ‘instructor technology issues.’ This category was defined as “the inability for instructors to have access to hardware and software” and we observed its impact on instructor ability to maintain *teaching presence*. For example, Roberto described how this barrier impacted him:

*Even with some of the adjustments—I’ve recorded some of my videos on Zoom and using a whiteboard or whatever. A lot of students are asking me, “Can you write a little better?” “I’m trying to do it a little better, but it’s hard.” Something that would take me to write something on a piece of paper or write something on a board is taking me 10 times longer. It’s 10 times more inefficient mainly because I don’t have the technology at home. ---Roberto*

Unlike Diane, Roberto encountered significant issues with technology in his teaching. He felt that his lack of technology at home created a barrier to providing his students with worthwhile educational experiences. Better access to technology would have allowed Roberto to improve his *teaching presence* for students. Here, we see the student Jax having trouble with technology:

*Unstable internet, unreliable technology, lack of motivation. –-Jax*

They described how lack of internet and unreliable technology led to their lack of motivation during class. As we aligned support and barrier categories with *teaching presence*, *social presence*, and other attributes, we discovered that there were more instances of *teaching presence* supports and barriers than the other two elements of the COI framework.

### Impact on *social presence*

*Social presence* is “the ability of learners to project their personal characteristics into the community of inquiry, thereby presenting themselves as ‘real people’”. The ‘community’ category within the theme of ‘mental and emotional support’ was something both students and instructors mentioned as important in their transition to ERT. Instructors discussed things like having a support group to allow them to learn from others and having friends to vent to about their frustrations. Having a sense of community provided a basis for *social presence* in that in our study we considered instructors as learners as well as students. Instructors in ERT were learning how to teach remotely in a pandemic; therefore, instructors’ *social presence* was also something important to consider in our analysis.

Another category that aligned with *social presence* was ‘student comfort interacting online,’ which was defined as “using communication tools and modalities familiar to students.”

*I found that the students were very engaged. It didn’t really puzzle them, this remote instruction to online. They were already prepared for it in other classes and the students are all doing very well in this class. ---Aarush*

Instructor Aarush described that his students’ familiarity with online classes allowed them to be more engaged, and thereby increased their *social presence*. However, other instructors saw a decrease in student *social presence* as the semester progressed:

*And then I guess, yeah, towards the end or towards the middle and the end of the semester, we started seeing less participation, except for those few students that were still engaged. Yeah. So I don’t know. It was crazy because, in the beginning, we’re like, all right, yeah, we got this kind of thing. But then at the end, people were like, ah. ---Katherine*

Instructor Katherine’s students’ enthusiasm and engagement decreased towards the end of the semester. This barrier to ERT was categorized as ‘student presence and participation*,’* which was defined as the “inability of students to attend or participate in class, office hours, or course activities.” We argue that lack of student presence and participation decreased student’s *social presence* during ERT.

### Impact on *cognitive presence*

*Cognitive presence* is defined as “the extent to which learners can construct and confirm meaning through sustained reflection and discourse.” The quality of *cognitive presence* reflects the quality and quantity of critical thinking, collaborative problem-solving, and construction of meaning occurring during student–student and student–instructor interactions (Garrison & Cleveland-Innes, 2005). The support theme ‘mental and emotional support’ was important, because poor mental health and distractions impacted motivation to explore course content, the first component of *cognitive presence*. For example, the support category ‘work from home’ allowed for personal support from spouses and pets, didn’t clearly indicate that there was an impact on *teaching* or *social presence*, but it clearly had an impact on instructors’ ability to teach during ERT and could impact their ability to support students’ *cognitive presence* in the classroom. For example, instructor Martin described how working from home allowed him to feel supported, “And, of course, informal support in mental issues, well, my spouse and my pets were really imperative to keep me sane*.*” Mental health, critical to both instructor and student success and *cognitive presence*, has been shown to play a significant role in the lives of many during the COVID-19 pandemic (Copeland et al., 2021; Kecojevic et al., 2020; Roman, 2020).

Many instructors found it difficult to manage a proper work–life balance as shown in the barrier category ‘instructor emotion,’ which was defined as “frustration, fatigue, and guilt due to remote delivery.” Instructor Claire described, “There were multiple days, where, if I did shower, it was at 3 in the afternoon and I was sobbing in the shower because I just couldn’t see it getting any better, and still try to maintain quality.” Claire’s struggle to maintain a separation between work and home life, coupled with her feelings of hopelessness, did not align with *teaching* or *social presence*, but might have impacted how she engaged with ERT, impacting students’ *cognitive presence*.

**Fig. 6:**
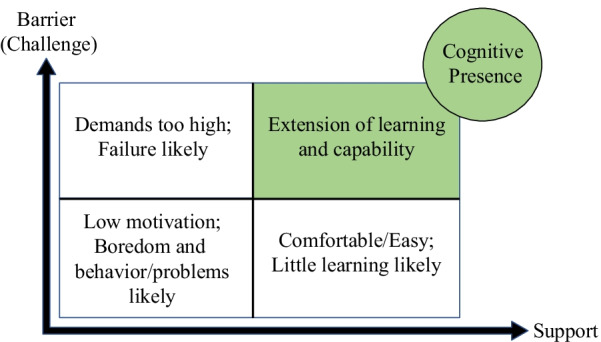
Impacts of scaffolding on *cognitive presence*. The upper left quadrant, ‘demands too high; failure likely,’ is when barriers or challenges are not met with equal support, leading to a likely failure for the student. The lower left quadrant, ‘low motivation; boredom and behavior/problems likely,’ is generally seen when challenges and supports are both too little. The lower right quadrant, ‘comfortable/easy; little learning likely,’ is when challenges are too low but supports are high. The upper right quadrant is when challenges or barriers and supports are equal, leading to an ‘extension of learning and capability’ Adapted from Hammond and Gibbons (2005)

In addition, knowing that the relationship between barriers (or challenges) and supports is important, we suggest that developing students’ *cognitive presence* through a balance of supports and barriers (or challenges) in *scaffolding* could lead to an extension of students’ learning and capabilities during ERT (Fig. 6).

## Discussion

Considering these supports and barriers in the COI and scaffolding frameworks, we found that some faculty found ways to maintain a strong *teaching presence* and a healthy work–life balance, but most of these supports were local to the instructor and existed prior to the transition to ERT. Despite institutional efforts, most of the broad, large-scale supports that faculty identified were logistical in nature and primarily helped the faculty to maintain a strong *teaching presence*. While faculty may not be familiar with the COI framework, they all recognized the importance of maintaining a strong *teaching presence* and facilitating a strong *social presence* for students. However, most of the faculty identified several barriers that prevented them from maintaining and facilitating *teaching* and *social presences*. In addition, most instructors had trouble maintaining a healthy work–life balance and found the transition to be extremely stressful, potentially impacting students’ *cognitive presence*. All of this suggests that either 1) the supports provided by the institution were not meeting the instructor and student needs and/or 2) the instructors and students were unaware of institutional supports.

None of the instructors or students identified institutional supports that helped them maintain a healthy work–life balance nor supported them in maintaining a strong *social presence* during ERT. Students at UC Merced are particularly at risk when it comes to pandemic-related barriers. The high number of students from marginalized backgrounds and first-generation families leave UC Merced students vulnerable to barriers other students might find easier to adjust to. Following this, recommendations for future emergencies for universities could include either more supports for instructors and students, such as continued access to free technology, both software and hardware, as well as increased access to reliable internet bandwidth or finding better ways to disseminate where and how to get these supports to instructors and students. In addition, maintaining a healthy work–life balance is vital for everyone. Increased workload was cited by many students and instructors as a significant struggle they faced. It is crucial that universities find ways to help alleviate this added strain, whether it be through hiring additional staff to create things such as laboratory instructional videos or simply lowering expectations, so that instructors and students that have family obligations directly stemming from the pandemic can attend to them. Additional time for transitions for preparation of new online course material or training for students to become more familiar with remote platforms would also be helpful in future ERT circumstances.

## Conclusions

In this article, we have examined the perceived supports and barriers that affected instructors and students during the rapid transition to ERT resulting from the COVID-19 pandemic. A total of 31 STEM instructors were interviewed about supports and barriers that they had experienced during the ERT transition, and interview transcripts were analyzed via two-cycle quantitative analysis process drawing from grounded theory methods. This process led to the identification of 134 unique supports that were collected into 12 categories, best represented by the themes (1) Tools and support for class content; (2) Mental/emotional support; and 3) Preexisting support. Similarly, instructors described 203 unique barriers that we collected into 14 categories, best represented by the themes (1) Tools and support for class; (2) Spending more time and effort; and 3) Affective issues. At the same time, 69 undergraduate students in STEM classes were surveyed about supports and barriers that they had experienced during the ERT transition. The student survey responses were analyzed by deductive coding, using the codes identified during the instructor analysis. The most frequent support categories identified by students were ‘course attributes,’ followed by ‘technology for remote teaching,’ and ‘community.’ The most frequent barrier categories identified by students fell into the ‘instructional space’ category, followed by the ‘student emotion and comfort’ category, and the ‘student presence and participation’ category. Finally, our data shows the importance of providing supports to enable instructors and students to maintain strong *teaching*, *social*, and *cognitive presence*. Without these supports, there are potential impacts on work–life balance and mental health of faculty and students.

### Limitations and future directions

We acknowledge that there are several factors that limit our study; however, these limitations provide opportunities for future studies. First, we conducted a convenience sample at only one MSI, UC Merced, so there is limited generalizability. In addition, we did not employ a systemic approach to ensure even distribution of faculty and students across STEM disciplines at our institutions, so there were more chemistry and biology participants based on the departments of the co-authors. In particular, the students surveyed were from only four of the 31 instructors interviewed. As the transition to ERT during the COVID-19 pandemic was a one-time event, we will not be able to collect more data from other MSIs or within UC Merced to see if our patterns persist in different study contexts or with more participants. It might be possible to work with other MSI’s that conducted similar work and compare our survey data for patterns. Second, this study focuses on self-report survey and interview data that is often perceived as less objective compared to well-developed and validated classroom observation protocols (American Association for the Advancement of Science [AAAS], 2012). A risk of surveys and interviews is that the participants inaccurately self-report their teaching and learning experiences, limiting the conclusions that can be made from these data. Our future work aims to triangulate the self-report survey and interview data with classroom observations and student learning gains data (i.e., concept inventories) to better understand classrooms interactions and student learning. Third, this study only described the perceived supports and barriers through the transition to ERT, not the continuation of it. Therefore, we collected instructor interview data at the end of the Fall 2020 semester to better understand what served as supports and barriers for instructors during the continuation of ERT. Taken together, we found there are more barriers than supports and that increased resources and communication between students, staff, faculty, and administration could help mitigate future emergency remote hardships and experiences.

## Supplementary Information

**Additional file 1.** Supplemental Tables 1–8.

## Data Availability

We have included Supplementary Material with some raw data and results. Additional data are available upon reasonable request and with permission of UC Merced’s Institutional Review Board.

## Abbreviations

CETL: Center for Engaged Teaching and Learning
COI: Community of Inquiry
DBER: Discipline-Based Education Research
ERT: Emergency Remote Teaching
IRB: Institutional Review Board
MSI: Minority-Serving Institution
SATAL: Students Assessing Teaching and Learning
STEM: Science, Technology, Engineering, and Mathematics
TA: Teaching Assistant
UCM: University of California, Merced
ZPD: Zone of Proximal Development
