# Correction to: I will teach you here or there, I will try to teach you anywhere: perceived supports and barriers for emergency remote teaching during the COVID-19 pandemic

**DOI:** 10.1186/s40594-022-00341-3

**Published:** 2022-03-14

**Authors:** Cristine Donham, Hillary A. Barron, Jourjina Subih Alkhouri, Maya Changaran Kumarath, Wesley Alejandro, Erik Menke, Petra Kranzfelder

**Affiliations:** 1 Department of Genetics, University of Georgia, Athens, GA USA; 2 Biology Teaching and Learning, University of Minnesota, Minneapolis, MN USA; 3 Cognitive and Information Sciences, University of California, Merced, CA USA; 4 Molecular and Cellular Biology, University of California Merced, Merced, CA USA; 5 Chemistry and Biochemistry, University of California, Merced, CA USA; 6 Department of Biology, Bemidji State University, Bemidji, MN USA; 7 Quantitative and Systems Biology Program, University of California, Merced, CA USA

## Correction to: International Journal of STEM Education (2022) 9:19 10.1186/s40594-022-00335-1

Following publication of the original article (Donham et al., 2022), the authors identified an error:

1. The wrong affiliation was listed for Jourjina Subih Alkhouri
  - Incorrect affiliation: Department of Genetics, University of Georgia, Athens, GA, USA.
  - Correct affiliation: Quantitative and Systems Biology Program, University of California, Merced, CA, USA.

The author group has been updated above and the original article has been corrected.
